# Microscopic Insights into Impurity-Modulated Capture of Platinum-Group Metals by Bismuth in Copper Anode Slimes

**DOI:** 10.3390/molecules31091383

**Published:** 2026-04-22

**Authors:** Dongji Liu, Hong Zeng, Fupeng Liu, Jing Cao, Huihui Xiong, Feixiong Chen, Tao Zhang, Jie Wang

**Affiliations:** School of Metallurgical Engineering, Jiangxi University of Science and Technology, Ganzhou 341000, China; liucyan19@163.com (D.L.); hongzeng2022@163.com (H.Z.); caoj2196@163.com (J.C.); 15579880267@139.com (F.C.); zt384385@163.com (T.Z.); wachen2019@163.com (J.W.)

**Keywords:** platinum group metals capture, bismuth metal, first-principles calculations, adsorption, impurities, microscopic mechanism

## Abstract

The efficient recovery of platinum group metals (PGMs) from decoppered anode slimes is essential for sustainable resource management, yet the atomic-level mechanisms underlying their capture remain unclear. Herein, first-principles calculations were employed to elucidate the microscopic interactions by which bismuth acts as a trapping agent for PGMs (Ru, Ir, Pt, Rh, Os, Pd) and to determine the effects of four representative impurities (As, Sb, Pb, Si). The results demonstrate that pristine Bi(001) exhibits strong chemisorption toward all six PGMs, as proved by the large charge transfer, significant electron sharing and pronounced p-d orbital hybridization. Furthermore, these impurities spontaneously incorporate into the Bi(001) surface due to the large binding energy. Crucially, some impurities such as As and Si function as potent surface activators rather than detrimental contaminants. These dopants significantly enhance the PGM binding strength by inducing intense localized charge redistribution and establishing strong orbital hybridizations among the Bi-5d, PGM-d and p orbitals of dopants. Overall, this work provides a theoretical foundation for strategically utilizing the impurities to optimize the recovery of PGMs in complex smelting systems.

## 1. Introduction

Platinum-group metals (PGMs) are indispensable in modern electronics, emerging energy technologies, and high-efficiency catalysis owing to their exceptional physicochemical properties [1,2,3]. Copper anode slime, a primary byproduct of the copper electrorefining process, is enriched with precious metals such as Au, Ag, and PGMs, while also containing environmentally hazardous heavy metals including Pd, As, and Cd [4,5]. Statistical data indicate that approximately 50% of Au, 60% of Ag, 35% of Pt, and 50% of Pd produced in China are derived from copper anode slimes [6,7,8,9]. While the massive generation of these slimes imposes considerable environmental pressure, it simultaneously presents promising opportunities for the recovery of valuable metal resources [10,11]. Conventional copper pyrometallurgical processes predominantly employ Pb as a collector for PGMs; however, this method is associated with high energy consumption and high As and Pb pollution. Recent studies have demonstrated that the recovery of bismuth (Bi) from high-bismuth copper anode slime enables the concurrent enrichment of Au, Ag, and PGMs, thereby confirming the feasibility and effectiveness of utilizing Bi to capture PGMs [12,13]. Consequently, an increasing number of studies have shifted their focus toward the eco-friendly metal Bi for the highly efficient capture of PGMs [1]. Nevertheless, the microscopic interactions and underlying bonding mechanisms between metallic Bi and PGMs during the collection process remain unclear.

Additionally, the composition of practical copper anode slime systems is inherently complex, inevitably containing various associated impurity elements such as As, Sb, Pb, and Si [14,15]. During high-temperature smelting, these impurities spontaneously migrate into the Bi melt, thereby altering the local lattice configuration and surface chemical environment [16]. Currently, the effects of these impurities on PGM collection efficiency, along with their potential micro-regulatory mechanisms, remain insufficiently understood [17]. Given that high-temperature metallurgical processes involve intricate multiphase reactions and mass transfer, conventional experimental techniques struggle to provide an in-depth resolution at the microscopic level. In recent years, first-principles calculations based on density functional theory (DFT) have proven highly effective in elucidating the adsorption behaviors of metal atoms on solid surfaces at the atomic scale, thereby uncovering the microscopic mechanisms of metal capture by adsorbents [18]. For example, in our recent work [19], we employed DFT to elucidate the microscopic mechanism of Au capture by bismuth in decoppered anode slimes, demonstrating that specific impurities can modulate the local electronic environment to influence Au adsorption. However, compared to the stable Au, PGMs possess highly active d orbitals, which leads to different bonding characteristics and higher sensitivity to local chemical coordination. Consequently, the mechanisms of Bi capturing PGMs, and how varying impurities modulate the complex orbital hybridizations across different PGM elements, cannot be simply extrapolated from previous study and remain unresolved.

Building upon these insights, first-principles calculations were employed to systematically investigate the adsorption behavior of six typical PGMs (Ru, Ir, Pt, Rh, Os, Pd) on both pristine and impurity-doped Bi(001) surfaces. Initially, a pristine Bi(001) surface model was constructed to evaluate its adsorption strength and bonding characteristics with the six PGMs. Subsequently, four impurity elements (As, Sb, Pb, Si) were introduced into the model. By calculating the binding energies, the thermodynamic stability of these impurities within the Bi lattice was analyzed, and the variations in PGM adsorption energies on the Bi(001) surface before and after impurity doping were comprehensively compared. Finally, electronic structure analyses, including differential charge density, electron density distribution, and projected density of states, were conducted to further reveal local charge redistribution induced by impurities and the multi-orbital hybridization characteristics among Bi, PGMs, and impurity atoms. The findings of this study provide a theoretical guidance for the rational utilization of associated impurities and optimization of PGM recovery processes in complex metallurgical environments.

## 2. Results and Discussions

### 2.1. Adsorption Behaviors of PGMs Atoms on Bi(001) Surface

**Figure 1:**
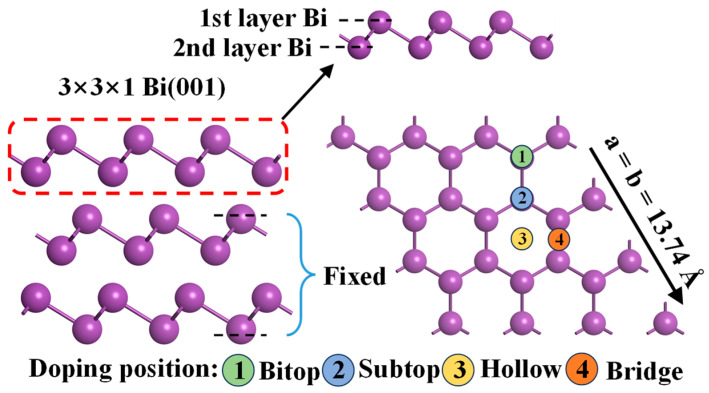
Geometrical configuration of (001) surface for the Bi metal, its potential adsorption sites and doping sites are also given.

**Figure 2:**
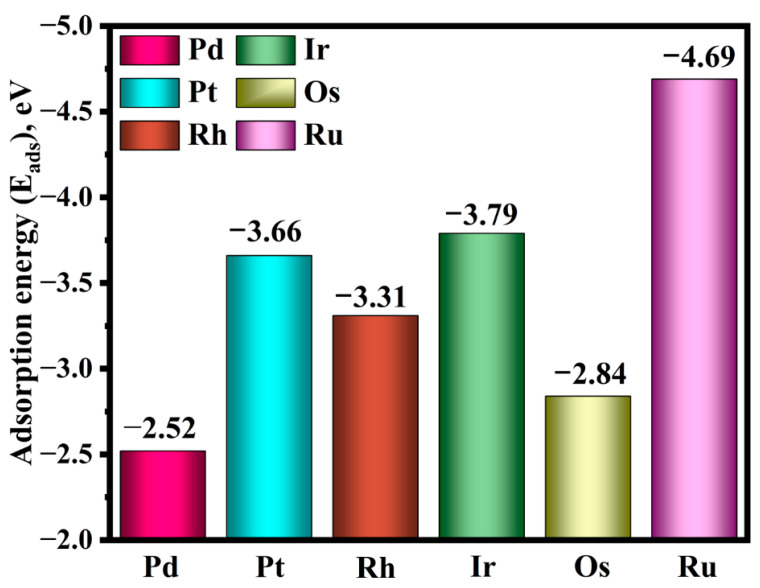
Calculated adsorption energies (*E*_ads_) of the six different PGM adatoms on the pristine Bi(001) surface.

Based on the pristine Bi(001) surface models (Figure 1), structural optimizations for the adsorption of the six PGMs at the four various binding sites (Bitop, Subtop, Hollow, and Bridge) were performed. After full relaxation, the adsorption energy of the lowest-energy structures for each PGM atom was determined. For clarity, these pristine adsorption systems are hereafter denoted as PGM/Bi(001). As shown in Figure 2 and Table 1, the calculated E_ads_ values for these PGMs atoms are all negative, spanning a substantial range from −2.52 eV to −4.69 eV. This indicates the exothermic and spontaneous nature of the PGM capture process by the bismuth metal. Notably, Bi(001) exhibits the strongest binding affinity toward the Ru with an E_ads_ of −4.69 eV. The interaction strength follows a decreasing sequence of Ru (−4.69 eV) > Ir (−3.79 eV) > Pt (−3.66 eV) > Rh (−3.31 eV) > Os (−2.84 eV) > Pd (−2.52 eV). The magnitudes of these adsorption energies confirm the formation of robust chemical bonds between the Bi surface and the PGM adatoms. This elucidates why the bismuth metal can serve as a highly efficient trapping agent for precious metals during the complex decoppered anode slime smelting process.

**Figure 3:**
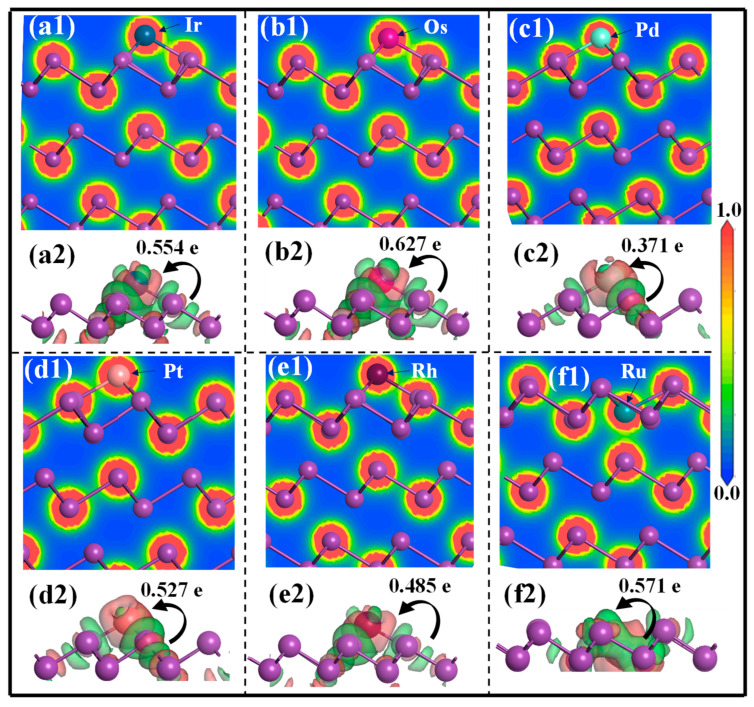
(**a1**–**f1**) Electron density distribution and (**a2**–**f2**) differential charge density of adsorbed Bi(001) surface with (**a1**,**a2**) Ir, (**b1**,**b2**) Os, (**c1**,**c2**) Pd, (**d1**,**d2**) Pt, (**e1**,**e2**) Rh, and (**f1**,**f2**) Ru. In the DCD plots, the red and green isosurfaces refer to the charge accumulation and charge depletion, respectively.

To reveal the microscopic origin of the strong binding affinities, the fully relaxed configurations of the PGM-adsorbed Bi(001) surface were thoroughly examined, as presented in Figure S1. One can see that the adsorption of PGM atoms induces localized structural reconstruction on the pristine Bi lattice. The bond lengths between the PGM adatoms and their nearest-neighbor Bi atoms ranges from 2.689 Å to 2.893 Å. These short atomic distances provide direct evidence for the formation of robust chemical bonds rather than weak physical interactions. The interfacial bonding characteristics were further elucidated by analyzing the differential charge density (DCD) and the corresponding 2D electron density distribution (EDD). As depicted in Figure 3(a2–f2), significant electron accumulation (red regions) surrounds the PGM adatoms, accompanied by an abundant electron depletion (green regions) over the adjacent Bi atoms. Quantitative charge analysis indicates that all the highly electronegative PGMs gain the electrons from the Bi substrate, with the net electron numbers of 0.371, 0.485, 0.527, 0.554, 0.571, and 0.627 e for Pd, Rh, Pt, Ir, Ru, and Os, respectively. These large charge transfer numbers again demonstrate the strong capture ability of Bi metal for the PGMs.

**Figure 4:**
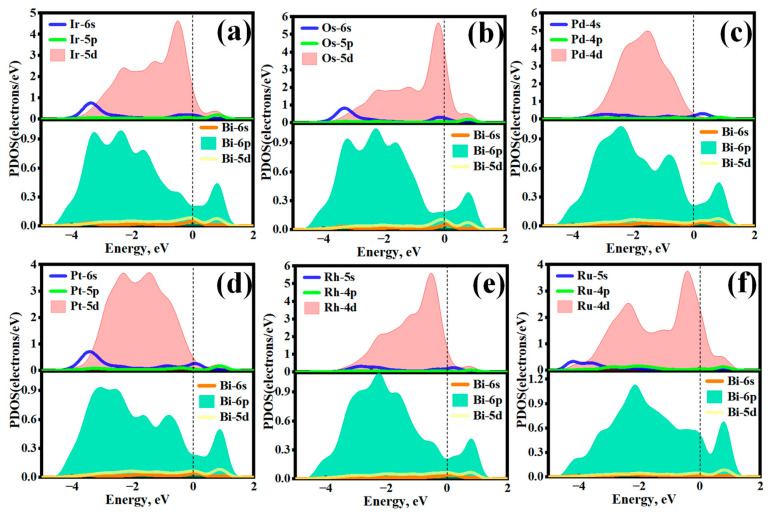
Projected density of states (PDOS) of the adsorbed Bi(001) surface with (**a**) Ir, (**b**) Os, (**c**) Pd, (**d**) Pt, (**e**) Rh, and (**f**) Ru. The dashed lines represent the Fermi level.

Additionally, the EDD maps in Figure 3(a1–f1) reveal significant electrons shared between the PGM adatoms and their adjacent Bi atoms, suggesting the formation of PGM-Bi metal bonds during the adsorption process. To further uncover the quantum mechanical fundamentals of this strong chemisorption, the projected density of states (PDOS) of each adsorbed system was calculated, as illustrated in Figure 4. Distinct orbital overlap can be observed between the Bi(001) surface and the respective PGM adatoms. Specifically, the localized d orbitals of the PGMs (4d for Pd/Rh/Ru; 5d for Ir/Os/Pt) exhibit pronounced resonance with the Bi-6p orbitals, particularly within the energy range from −6.0 eV to the Fermi level. The broad and continuous distribution of these resonant peaks indicates robust p–d orbital hybridization. This strong orbital coupling, together with significant charge transfer, underpins the high binding stability of PGMs on Bi(001), thereby establishing the microscopic mechanism for the efficient capture of precious metals by bismuth in copper anode slimes.

### 2.2. Influence of Impurities on the Adsorption Properties of PGM Atoms

#### 2.2.1. Stability Analysis of Doped Bi(001)

Before investigating the effects of impurities on the PGM capturing behavior, it is imperative to ascertain the thermodynamic stability of these dopant elements (DEs, DEs = As, Pb, Sb, Si) decorating Bi(001). The structural stability of these doped Bi(001) elements can be evaluated via the binding energy (*E*_bin_), which is defined as [19,20]:

(1) $$E_{bin}=E_{DE-Bi(001)}-E_{Bi\left(001\right)-vac}-E_{DE}$$

where $E_{DE-Bi(001)}$ represents the total energy of the Bi(001) surface doped with a DE, $E_{Bi\left(001\right)-vac}$ denotes the energy of the defective Bi(001) surface with a single Bi vacancy, and $E_{DE}$ is the energy of a single dopant atom.

**Figure 5:**
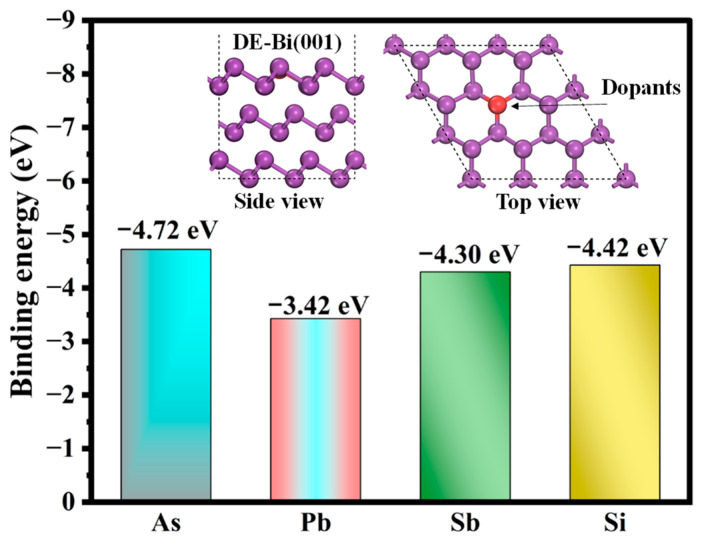
Calculated binding energies (E_bin_) for the incorporation of various dopant elements (As, Pb, Sb, Si) into the Bi(001) surface via atomic substitution.

Under this definition, the binding energy assesses whether the DEs can stably reside within the Bi(001), and a negative *E*_bin_ signifies an energetically favorable and exothermic atomic substitution process. Figure 5 gives a comparison in the binding energies of four Des with doped Bi(001). The calculated *E*_bin_ values for all impurity atoms are consistently negative, demonstrating a strong thermodynamic driving force for their spontaneous incorporation into the Bi vacancy sites. The binding stability follows a decreasing sequence of As (−4.72 eV) > Si (−4.42 eV) > Sb (−4.30 eV) > Pb (−3.42 eV). These large negative *E*_bin_ values indicate the formation of highly stable doped structures that resist spontaneous phase segregation. Notably, As, Si, and Sb are more readily anchored within the Bi lattice compared to Pb. Consequently, these doped systems are selected for further investigation of the PGM capture performance of Bi metal.

#### 2.2.2. Effect of As, Sb, Pd and Si Doping

**Figure 6:**
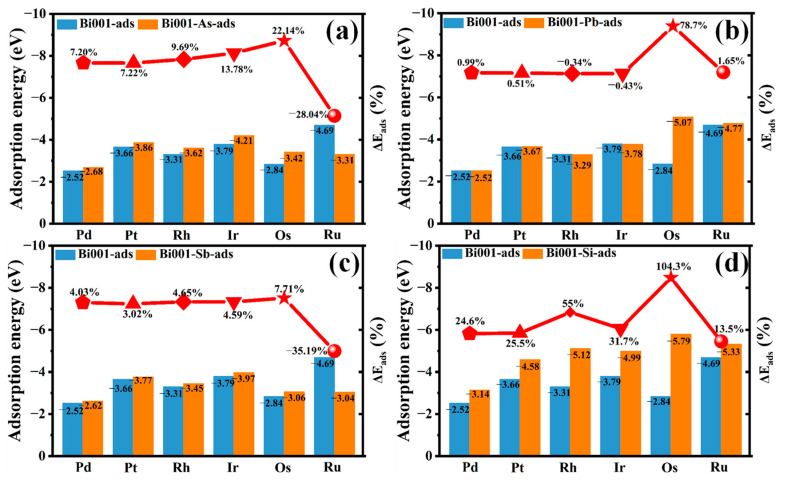
Effect of impurities doping on the adsorption strength of PGMs’ adsorbed Bi(001) surface: (**a**) As, (**b**) Pb, (**c**) Sb, and (**d**) Si.

To evaluate how the DEs modulate the binding affinity of Bi(001) toward PGMs, we conduct a comprehensive comparison of the adsorption energy (*E*_ads_) and the corresponding relative variation ratios (Δ*E*_ads_) between the pristine and impurity-doped Bi(001). As depicted in Figure 6a,d and Table S1, the incorporation of As and Si into the Bi lattice enhances the interaction strength with nearly all PGMs, resulting in significantly more negative E_ads_ values. This enhancement is most dramatic in the Si-doped Bi(001) system (Figure 6d), which exhibits exceptional increases in binding affinity, with E_ads_ improving by 104.3% for Os and 55% for Rh. Similarly, As doping activates the local chemical environment, yielding notable E_ads_ enhancements of 13.78% for Ir and 22.14% for Os. These substantial positive increments in ΔE_ads_ indicate that these impurities effectively increase the thermodynamic driving force for PGM adsorption, making the capture process energetically more favorable.

In contrast, Sb doping exerts a minor or even adverse effect on the capture process (Figure 6c). Specifically, Sb doping results in a large negative ΔE_ads_ of −35.19% for Ru, indicating a significant detrimental impact on Ru capture. Pb doping shows a negligible effect overall, with ΔE_ad_s values fluctuating around zero (mostly within ±2%) except for Os capture, where Pb doping markedly improves adsorption, yielding a ΔE_ads_ of 78.70% (Figure 6b). These findings suggest that Sb and Pb impurities generally fail to induce the electronic perturbations necessary to strengthen Bi–PGM bonds, with the notable exception of Pb enhancing Os adsorption. From a macroscopic metallurgical perspective, these microscopic insights are highly informative. Compared to the conventional Pb-based collection process, which is limited by severe environmental and health hazards [11,21], recent experimental studies [1,12,13] have demonstrated that high-bismuth melts exhibit superior collection efficiency for precious metals. Our calculated adsorption energies (ranging from −2.52 eV to −4.69 eV) indicate that the capture ability of bismuth melts is significantly stronger than that of both Pb melts and copper matte. Moreover, the subsequent electronic property analyses provide the atomic-level validation for these experimental observations, confirming the thermodynamic feasibility of substituting Pb with environmentally friendly Bi.

Additionally, the regulatory role of impurities in this work provides a deeper and more complex perspective compared to existing theoretical literature. In our previous investigation regarding Au capture by Bi [19], we found that impurities modulated Au adsorption primarily through relatively simple electronic perturbations. Similarly, Huang et al. [22] reported that both As and Sb dopants enhance the gold-capturing ability of Cu_2_S. In contrast, the present findings reveal impurity-dependent behavior for PGMs due to their active d orbital configurations. While As and Si act as potent activators for PGM capture, Sb exhibits a largely detrimental effect, such as decreasing Ru adsorption by 35.19%. This different response highlights that the orbital hybridization in PGM systems is more sensitive to the dopant type than in Au systems. Consequently, the presence of As and Si impurities in decoppered anode slimes is not detrimental; rather, these elements act as activators that facilitate more robust and efficient trapping of precious metals. This comparison underscores important industrial guidance: the complete removal of all associated impurities prior to smelting is energy-intensive and unnecessary. Instead, retaining some specific impurities like As and Si can optimize the PGM recovery in complex copper smelting processes.

#### 2.2.3. Electronic Properties Analysis

To elucidate the microscopic mechanisms of the enhanced PGM adsorption induced by DE doping, six representative adsorption systems, denoted as PGMs@DE-Bi(001), are selected for in-depth electronic structure analysis. Figure S2 presents the fully relaxed atomic configurations of Ir@As-Bi(001), Os@As-Bi(001), Os@Pb-Bi(001), Pb@Si-Bi(001), Pt@Si-Bi(001), and Ir@Si-Bi(001) adsorption systems. It is evident that the introduction of impurity atoms alters the local coordination environment of the Bi(001) surface. Furthermore, the PGM adatoms in these doped structures preferentially anchor themselves at the hollow sites, forming chemical bonds with both the host Bi atoms and the adjacent dopants. To further reveal the nature of this interaction, these distances are compared to the sum of their respective van der Waals (vdW) radii. The calculated bond lengths (d_Ir−As_ = 2.346 Å, d_Os−As_ = 2.328 Å, and d_Pt−Si_ = 2.284 Å) are obviously smaller than the sum of their corresponding vdW radii (~3.85 Å for Ir/Os and As, and ~3.85 Å for Pt and Si). This significant reduction in atomic distance indicates that these dopants act as highly active sites that facilitate the formation of strong covalent bonds.

**Figure 7:**
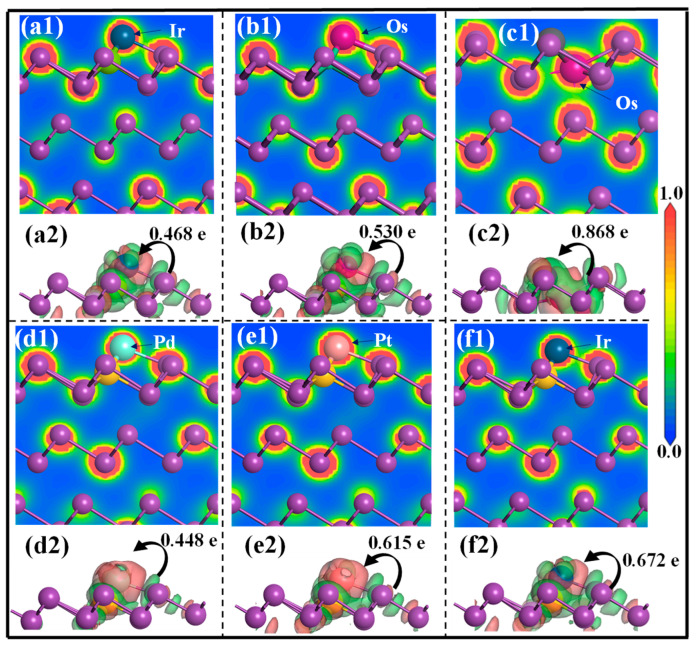
(**a1**–**f1**) Electron density distribution and (**a2**–**f2**) differential charge density of various adsorption systems: (**a1**,**a2**) Ir@As-Bi(001), (**b1**,**b2**) Os@As-Bi(001), (**c1**,**c2**) Os@Pb-Bi(001), (**d1**,**d2**) Pd@Si-Bi(001), (**e1**,**e2**) Pt@Si-Bi(001), (**f1**,**f2**) Ir@Si-Bi(001).

To further elaborate the nature of this enhanced adsorption effect, the DCD and EDD plots for different adsorption systems are calculated, as shown in Figure 7. In Figure 7(a2–f2), significant electron accumulation is strongly localized in the interstitial regions bridging the PGMs and the impurity/Bi active sites, while pronounced electron depletion occurs around the top two layers of Bi atoms. As a result, the PGM adsorbates act as electron acceptors, acquiring approximately 0.468 e, 0.530 e, 0.868 e, 0.448 e, 0.615 e, and 0.615 e from the doped Bi(001) surface. This substantial charge transfer serves as a primary electrostatic driving force, underpinning the marked enhancement in PGM capture efficiency induced by impurity doping. Moreover, the EDD maps in Figure 7(a1–f1) reveal dense and continuous electron density accumulation between the PGM adatoms and their adjacent Bi and dopant atoms (As, Pb, and Si). This pronounced electron sharing indicates the formation of strong metallic and covalent bonds, providing a mechanistic explanation for the improved recovery efficiency of PGMs by bismuth metal in copper anode slimes containing specific impurities.

**Figure 8:**
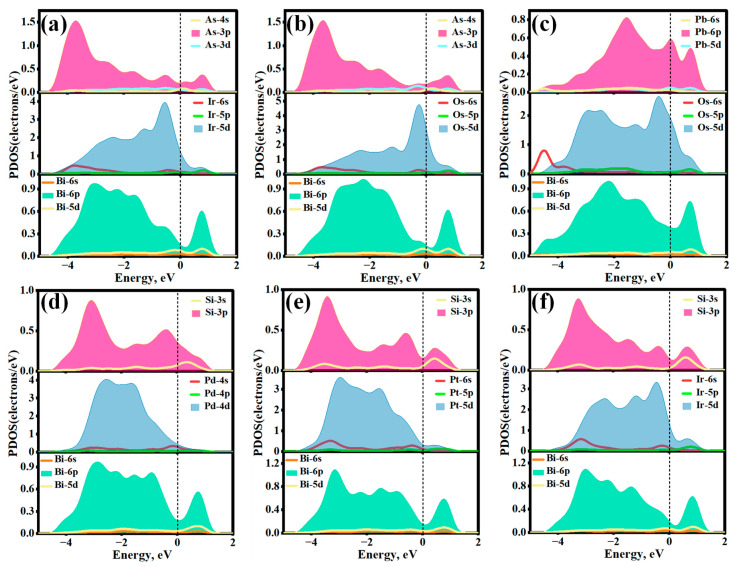
Projected density of states (PDOS) of various adsorption systems: (**a**) Ir@As-Bi(001), (**b**) Os@As-Bi(001), (**c**) Os@Pd-Bi(001), (**d**) Pd@Si-Bi(001), (**e**) Pt@Si-Bi(001), (**f**) Ir@Si-Bi(001). The dashed lines represent the Fermi level.

To elucidate the underlying bonding mechanisms, the PDOS for the aforementioned six adsorption systems was systematically analyzed, as presented in Figure 8. In all cases, the highly localized d orbitals of the PGMs (5d for Ir, Os, Pt and 4d for Pd) exhibit strong and continuous resonance with the Bi-6p orbitals, primarily within the energy range of −4.50 eV to the Fermi level. For example, in the Ir@As-Bi(001) and Os@As-Bi(001) systems (Figure 8a,b), the Ir-5d and Os-5d orbitals strongly hybridize with the As-4p and Bi-6p states, producing significant, broad resonance peaks around −2.5 to −1.5 eV. Additionally, the d orbitals of the PGMs interact strongly with the outermost p orbitals of the dopants (As, Pb, and Si) over the range of −4.50 eV to 1.80 eV. In the Pd@Si-Bi(001) and Pt@Si-Bi(001) systems (Figure 8d,e), pronounced hybridizations between Si-3p states and Pd/Pt-d states give rise to several resonance peaks near −2.50 eV and −1.50 eV. In conclusion, the incorporation of impurities induces robust ternary p–d orbital hybridization (host–PGM–dopant), thereby enhancing the binding strength of Bi(001) toward PGMs and enabling highly efficient trapping of these metals.

## 3. Calculation Method and Details

### 3.1. Calculation Details

All first-principles simulations were executed utilizing the DMol^3^ code integrated within the BIOVIA Materials Studio 2020 (Dassault Systèmes BIOVIA, San Diego, CA, USA) [23]. To accurately handle electron-exchange and correlation interactions, the Perdew–Burke–Ernzerhof (PBE) formulation under the generalized gradient approximation (GGA) was adopted [24,25]. The electronic wave functions were expanded utilizing a double-numerical plus polarization (DNP) basis set with an orbital cutoff of 5.2 Å [26]. Furthermore, the DFT semi-core pseudopotential (DSPP) was applied to effectively replace core electrons and capture the relativistic effects inherent in heavy metal elements [27]. Given the heavy atomic masses of Bi and PGMs, spin–orbit coupling (SOC) effects were considered. Nevertheless, preliminary tests indicated that SOC barely alter the results and was therefore omitted in this work. K-points meshes of 4 × 4 × 1 using Monkhorst–Pack scheme [28] were assigned for optimizing atomic coordinates, whereas denser 9 × 9 × 1 grids were chosen for evaluating detailed electronic characteristics. Furthermore, the convergence thresholds of the maximum energy, force on atoms, and atomic displacement are 1.0 × 10^−5^ Ha, 0.002 Ha/Å, and 0.005 Å, respectively. To prevent artificial electrostatic coupling arising from periodic boundary conditions, a 15 Å vacuum thickness was inserted normal to the slab configurations. Finally, the affinity of the Bi(001) surface towards the platinum group of metals (PGMs) was assessed by calculating the adsorption energy (*E*_ads_), which is defined as [29]:

(2) $$E_{\mathrm{ads}}=E_{\mathrm{PGMs}/\mathrm{Bi}(001)}-E_{\mathrm{Bi}(001)}-E_{\mathrm{PGMs}}$$

Herein, $E_{\mathrm{PGMs}/\mathrm{Bi}(001)}$, $E_{\mathrm{Bi}(001)}$, and $E_{\mathrm{PGMs}}$ are the energies of PGM adsorbed systems, pristine Bi(001) slab, and a single PGM atom, respectively.

### 3.2. Structural Models

To systematically investigate the microscopic capture mechanisms of PGMs by bismuth metal, a series of structural models were established. First, a 3 × 3 × 1 supercell model of the pristine Bi(001) surface was constructed. During the structural optimization, the bottom four atomic layers of the slab were constrained at their optimized bulk coordinates to simulate the bulk phase, while the uppermost layers and all adsorbates were allowed to fully relax. For the PGM adsorption models, a single PGM atom (Ru, Ir, Pt, Rh, Os, or Pd) was placed on the surface. To identify the most stable configurations, four potential possible adsorption sites on the Bi(001) surface were evaluated: the top site of the first-layer Bi atom (Bitop), the top site of the second-layer Bi atom (Subtop), the threefold hollow site (Hollow), and the bridge site connecting two adjacent surface Bi atoms (Bridge), as illustrated in Figure 1. Furthermore, to evaluate the effects of impurities on PGM capture, impurity-doped Bi(001) models were constructed. Four representative impurity elements (As, Sb, Pb, and Si) were introduced into the system by substituting a top-layer surface Bi atom. The adsorption behaviors of the six PGMs on these impurity-decorated surfaces were subsequently investigated and comprehensively compared with those on the pristine Bi(001) surface.

## 4. Conclusions

First-principles calculations were conducted to elucidate the microscopic mechanisms underlying the capture of PGMs by bismuth metal, as well as the effects of four typical impurities (As, Sb, Pb, Si) present in copper anode slimes. The results indicate that pristine Bi(001) exhibits spontaneous and strong chemisorption affinity toward all six PGMs, following the interaction sequence Ru > Ir > Pt > Rh > Os > Pd. These robust interactions are primarily attributed to significant electron transfer and strong p–d orbital hybridization between the PGMs and Bi atoms. Binding energy calculations demonstrate that the four impurity elements energetically favor stable incorporation into the Bi lattice, thereby altering the local coordination environment. Importantly, comparative adsorption analyses identify As and Si dopants as effective activators rather than detrimental impurities, significantly enhancing the binding strength for PGM adsorption. Electronic structure analyses further confirm that this enhancement arises from intense charge redistribution and the formation of strong ternary host–PGM–dopant orbital hybridizations. These findings not only offer atomic-level validation for the experimental feasibility of utilizing eco-friendly Bi as a highly efficient substitute for PGM recovery, but also provide theoretical guidance for optimizing precious metal recovery by strategically using specific impurities in complex smelting systems.

## Figures and Tables

**Table 1:** Adsorption energy (E_ads_), the nearest-neighbor distances between the PGM atoms and the surface Bi atom (d_PGM-Bi_), and electron transfer (Δ*Q*) of PGMs adsorbed on the Bi(001).

Adsorbate	E_ads_, eV	d_PGM-Bi_, Å	Δ*Q*, e
Pd	−2.52	2.813	0.371
Pt	−3.66	2.771	0.527
Rh	−3.31	2.716	0.485
Ir	−3.79	2.702	0.554
Os	−2.84	2.689	0.627
Ru	−4.69	2.883	0.571

## Data Availability

The original contributions presented in this study are included in the article/Supplementary Materials. Further inquiries can be directed to the corresponding authors.

## Supplementary Materials

The following supporting information can be downloaded at: https://www.mdpi.com/article/10.3390/molecules31091383/s1, Figure S1: The lowest-energy structures of different PGMs atoms adsorbed on pristine Bi(001) surface; Figure S2: The most stable structures for different adsorption systems; Table S1: Adsorption energies (E_ads_) and relative variation ratios (ΔE_ads_) of PGMs on pristine and impurity-doped Bi(001) surfaces.
