# Supplementary material for: Microscopic Insights into Impurity-Modulated Capture of Platinum-Group Metals by Bismuth in Copper Anode Slimes
Source: Molecules. 2026 Apr 22;31(9):1383. doi: 10.3390/molecules31091383 (PMC13165233; doi:10.3390/molecules31091383)
Supplement: Supplementary file 1 [file molecules-31-01383-s001.zip › molecules-4250949-supplementary.pdf]

Supporting Information

Microscopic Insights into Impurity-Modulated Capture of Platinum-Group Metals by Bismuth in Copper Anode Slimes

Dongji Liu, Hong Zeng, Fupeng Liu *, Jing Cao, Huihui Xiong *, Feixiong Chen, Tao Zhang and Jie Wang

School of Metallurgical Engineering, Jiangxi University of Science and Technology, Ganzhou 341000, China; liucyan19@163.com (D.L.); hongzeng2022@163.com (H.Z.); caoj2196@163.com (J.C.); 15579880267@139.com (F.C.); zt384385@163.com (T.Z.); wachen2019@163.com (J.W.)

* Correspondence: fupengliu@126.com (F.L.); xionghui8888@126.com (H.X.)

Contents

Figure S1 The lowest-energy structures of different PGMs atoms adsorbed on pristine Bi(001) surface: (a) Ir/Bi(001), (b) Os/Bi(001), (c) Pd/Bi(001), (d) Pt/Bi(001), (e) Rh/Bi(001), and (f) Ru/Bi(001).

Figure S2 The most stable structures for different adsorption systems: (a) Ir@As-Bi(001), (b) Os@As-Bi(001), (c) Os@Pb-Bi(001), (d) Pb@Si-Bi(001), (e) Pt@Si-Bi(001), (f) Ir@Si-Bi(001).

Table S1 Adsorption energies (E_{ads}) and relative variation ratios (ΔE_{ads}) of PGMs on pristine and impurity-doped Bi(001) surfaces.

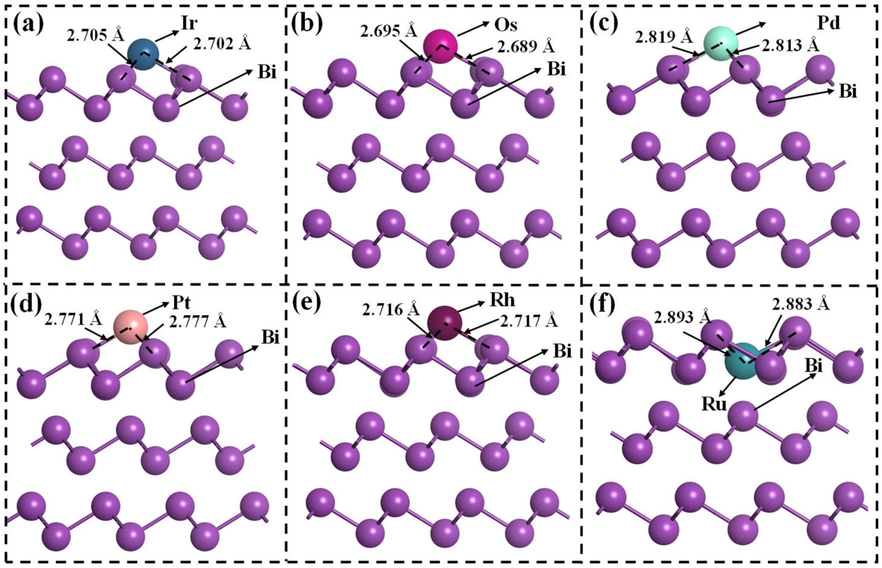

Figure S1 The lowest-energy structures of different PGMs atoms adsorbed on pristine Bi(001) surface: (a) Ir/Bi(001), (b) Os/Bi(001), (c) Pd/Bi(001), (d) Pt/Bi(001), (e) Rh/Bi(001), and (f) Ru/Bi(001).

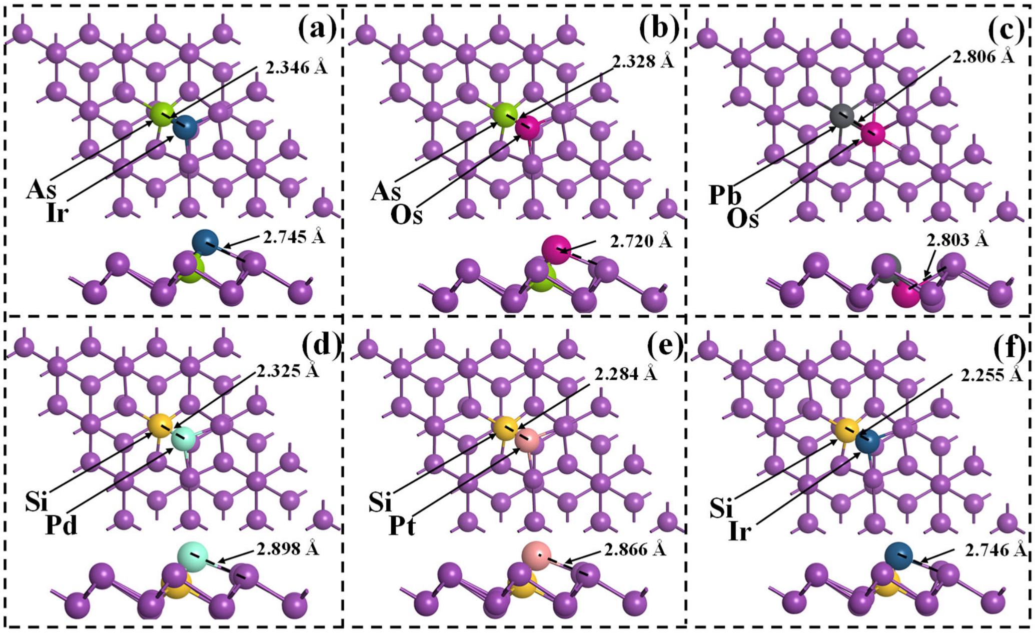

Figure S2 The most stable structures for different adsorption systems: (a) Ir@As-Bi(001), (b) Os@As-Bi(001), (c) Os@Pb-Bi(001), (d) Pb@Si-Bi(001), (e) Pt@Si-Bi(001), (f) Ir@Si-Bi(001).

Table S1 Adsorption energies (E_{ads}, eV) and relative variation ratios (ΔE_{ads}, %) of PGMs on pristine and impurity-doped Bi(001) surfaces.

Adsorbate	Bi(001)	As-Bi(001)		Pb-Bi(001)		Sb-Bi(001)		Si-Bi(001)	
	E_{ads}	E_{ads},	ΔE_{ads}	E_{ads}	ΔE_{ads}	E_{ads}	ΔE_{ads}	E_{ads}	ΔE_{ads}
Pd	−2.52	−2.68	7.20 %	−2.52	0.99 %	−2.62	4.03 %	−3.14	24.60 %
Pt	−3.66	−3.86	7.22 %	−3.67	0.51 %	−3.77	3.02 %	−4.58	25.50 %
Rh	−3.31	−3.62	9.69 %	−3.29	−0.34 %	−3.45	4.65 %	−5.12	55.00 %
Ir	−3.79	−4.21	13.78 %	−3.78	−0.43 %	−3.97	4.59 %	−4.99	31.70 %
Os	−2.84	−3.42	22.14 %	−5.07	78.70 %	−3.06	7.71 %	−5.79	104.30 %
Ru	−4.69	−3.31	−28.04 %	−4.77	1.65 %	−3.04	−35.19 %	−5.33	13.50 %
